# Reteplase versus Streptokinase in Management of ST-segment Elevation Myocardial Infarction; a Letter to the Editor

**DOI:** 10.22114/ajem.v0i0.143

**Published:** 2019-04-15

**Authors:** Fahimeh Giahchi, Mahmonir Mohammadi

**Affiliations:** 1. Department of Cardiology, Faculty of Medicine, Tehran Medical Sciences, Islamic Azad University, Tehran, Iran.

Although the ideal treatment route for management of patients with ST-segment elevation myocardial infarction (STEMI) is rapid diagnosis and direct transfer to the catheterization unit and undergoing primary angioplasty, using thrombolytic still has its place in cases that the equipment are not available or there is not enough time for performing angioplasty (1, 2). Of course these drugs are associated with specific side effects such as probability of gastrointestinal (GI) and cerebral bleeding, allergic reactions and etc. (2). Therefore, finding drugs with fewer side effects and limitations for use has always been interesting in this regard. The drug which has been used all around the world since 1970 and is also traditionally used in Iran for managing patients with STEMI is streptokinase. This drug is a protein extracted from beta hemolytic streptococci, which combines with plasminogen and facilitates transformation of plasminogen to plasmin. However, recently fibrin specific drugs or recombinant tissue plasminogen activators (rtPA) such as alteplase, urokinase, tenecteplase, reteplase and etc. have become available to emergency medicine physicians and cardiologists (3). Having longer half-lives, fewer side effects, and easier method of use, these drugs have opened a new door for physicians regarding use of thrombolytic drugs in treatment of STEMI and brain stroke. Studies regarding comparison of safety and effectiveness of these drugs are ongoing. In a systematic review and meta-analysis, Tourani et al. showed that the safety and effectiveness of streptokinase and tenecteplase were in the same level (4).

Reteplase is an rtPA peptide, which converts endogenous plasminogen to plasmin. Plasmin causes destruction of the fibrin present in the clot and the clot disappears. In a prospective case-control study in Amiralmomenin Hospital, Tehran, Iran, we divided 152 patients with STEMI and the mean age of 56.41 ± 10.40 (27 – 85) years who were candidates for receiving thrombolytic therapy into 2 groups receiving either streptokinase (from CSL Behring GmbH Co, Germany) or reteplase (from Reliance Life Sciences Co, India) (83.6% male). Then we compared outcomes such as GI bleeding, mortality, hypotension, arrhythmia, and etc. between the 2 groups. The 2 groups were in a similar condition regarding sex (p = 0.331) and age distribution (p = 0.393), blood pressure on admission (p = 0.378), and the rate of positive troponin on admission (p = 0.113). Overall, 61 (40.1%) patients showed at least one of the outcomes that we studied (13 cases in the streptokinase group and 48 cases in the reteplase group; p < 0.0001). In this study, all 20 cases of GI bleeding observed following thrombolytic prescription were in the reteplase group. Additionally, out of the 19 cases of death observed, 14 were in reteplase group and 5 had received streptokinase. One case of arrhythmia and 2 cases of hypotension were seen in streptokinase group. Based on the findings of this study, it seems that despite factors such as longer serum half-life and ease of use, the rate of side effects of this drug should be carefully considered before use. Based on some existing studies, it seems that in the most optimistic scenario, these 2 groups have similar side effects and effectiveness (4, 5).

For a more accurate assessment at least regarding the Iranian race, more accurate studies with larger sample size are required. This topic might have received less attention in developed countries as considering availability of equipment in those countries, treatment protocols are mostly based on angioplasty and not using thrombolytic drugs there. This subject becomes more important when we are aware of the higher treatment costs of these new drugs for patients since insurance does not cover them. Performing cost/benefit studies and evaluating the safety of treatment are suggested for future studies.
